# A dominant insulin-specific and islet-destructive T-cell response is sufficient to activate CD8 T cells directed against the fatty-acid receptor GPR40

**DOI:** 10.1038/s41423-019-0309-y

**Published:** 2019-10-24

**Authors:** Andreas Spyrantis, Jana Krieger, Katja Stifter, Bernhard Otto Boehm, Reinhold Schirmbeck

**Affiliations:** 1 Department of Internal Medicine I, Ulm University Hospital; Albert Einstein Allee 23, 89081 Ulm, Germany; 2 Lee Kong Chian School of Medicine, Nanyang Technological University, Singapore, Singapore; 3 Imperial College London, London, UK

**Keywords:** Autoimmunity, Adaptive immunity, Mechanisms of disease

Type 1 diabetes mellitus (T1D) is an autoimmune disease that is characterized by a progressive infiltration of autoreactive T cells into the pancreatic islets and the destruction of insulin-producing beta cells.^1^ It is generally assumed that T1D is initiated by yet unidentified T cells that escape from thymic negative selection^2^ and trigger an initial destruction of beta cells.^3^ These initial hits could generate suitable conditions in beta cells and/or in islets that favor the coactivation and amplification of autoreactive T cells directed against a broad spectrum of beta cell-specific antigens, such as GAD65, IGRP, and IA-2.^1,4^

The expression/processing of beta cell antigens in the endoplasmic reticulum (ER) can increase the presentation efficacy of epitopes that bind MHC class I molecules with low affinity.^2^ We showed that a preproinsulin (ppins)/(K^b^/A_12–21_) epitope with a very low affinity for K^b^ molecules efficiently induces K^b^/A_12–21_-specific CD8 T cells and diabetes in RIP-B7.1 mice (mice that express the costimulatory molecule B7.1 in beta cells), in coinhibition-deficient PD-L1^−/−^ mice and in anti-PD-L1-treated wild-type C57BL/6 J (B6) mice, when  various vector-encoded ppins designer antigens are expressed in the ER but not in the cytosol/nucleus.^5,6^ Using bone marrow chimeric mice, we confirmed that both a deficiency of PD-L1 in somatic target cells and/or a deficiency of PD-1 in T cells allows the induction of autoreactive ppins/(K^b^/A_12–21_)-specific CD8 T cells by DNA immunization.^6^ PD-L1 expressed on beta cells thus plays a crucial gatekeeper function to maintain self-tolerance and prevent autoimmune diabetes through ppins/(K^b^/A_12–21_)-specific CD8 T cells.^6^ In contrast, autoimmune diabetes can be induced in RIP-B7.1 mice, but not in PD-L1^−/−^ or in anti-PD-L1-treated B6 mice, by ppins/(K^b^/B_22–29_)-specific CD8 T cells that are directed against a high-affinity ppins/(K^b^/B_22–29_) epitope and exclusively primed by a mutant ppinsΔA_12–21_ antigen (lacking the K^b^/A_12–21_ epitope).^6,7^ Ppins/(K^b^/B_22–29_)-specific CD8 T cells critically depend on ‘help' from coprimed ppins/(K^b^/A_12–21_)-specific CD8 T cells to expand and develop their diabetogenic IFNγ^+^ effector phenotype^8^ in PD-L1-deficient mice.^6,7^ Ppins/K^b^/A_12–21_-specific CD8 T cells are thus a prototype of immunodominant autoreactive CD8 T cells that can trigger initial hits in beta cells in PD-L1^−/−^ mice.

An interesting source of beta cell antigens that can access various MHC I processing/presentation pathways are membrane-anchored proteins that contain transmembrane helices (TMHs) with multiple hydrophobic residues for spanning membranes.^9^ Bioinformatics analysis predicted an overrepresentation of TMHs among strong, high-affinity MHC class I binding epitopes,^9^ which therefore represent a large antigen repertoire for targeting high-affinity CD8 T cells. To confirm this, we chose a murine-free fatty acid receptor 1 (GPR40; Fig. 1a) that is expressed in murine and human beta cells.^10^ Indeed, a single injection of pCI/GPR40, but not of empty pCI DNA into RIP-B7.1 mice, induced hyperglycemia and autoimmune diabetes (Fig. 1b). Hyperglycemia was reversed in pCI/GPR40-immune diabetic RIP-B7.1 mice (with blood glucose levels between 370 and 400 mg/dl) by two consecutive injections of anti-CD8 antibodies, but not anti-CD4 antibodies (Fig. 1c).^6^ In line with this finding, diabetes development was characterized by a continuous infiltration of islets by CD8 T cells, a concomitant destruction of beta cells and decreased production of insulin (Fig. 1d). CD8 T cells were thus crucial for GPR40-induced diabetes in RIP-B7.1 mice.

**Fig. 1:**
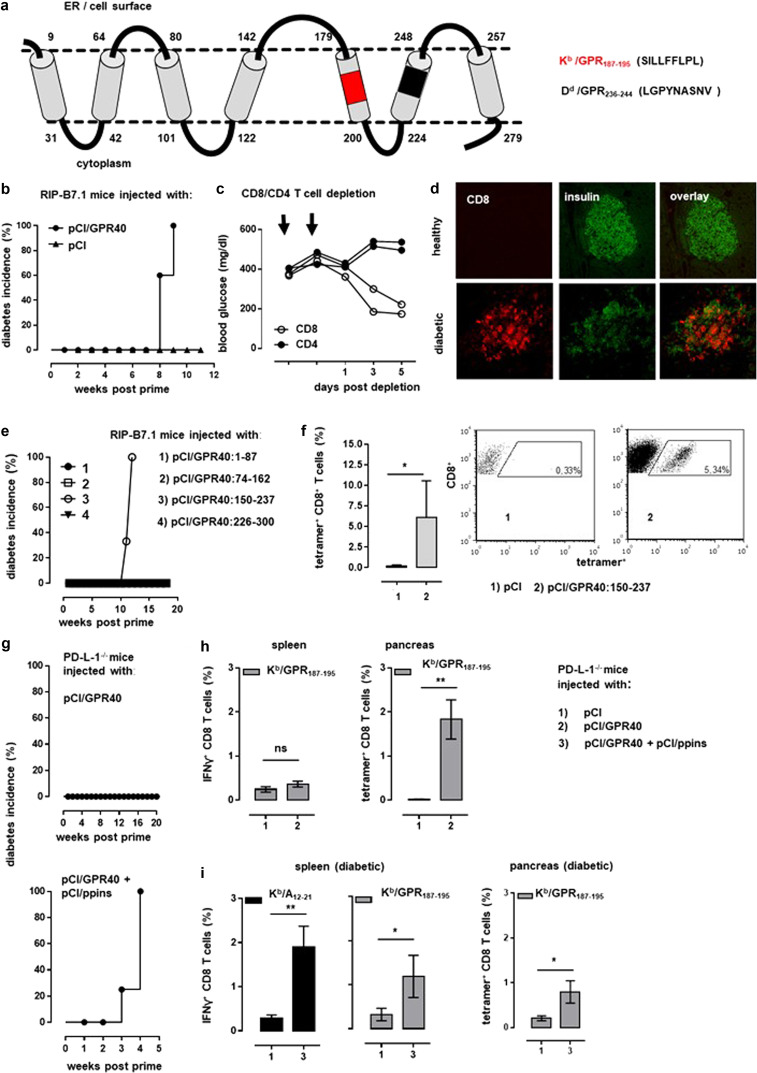
**a** Illustration of murine GPR40 and its seven transmembrane domains (swissprot. acc. no: Q76JU9). In addition, the localization of the newly identified K^b^/GPR40_187-195_ and D^d^/GPR40_236–244_ epitopes in TMHs is shown. **b** RIP-B7.1 mice were injected with pCI (triangles; *n* = 5) or pCI/GPR40 DNA (circles; *n* = 5), and blood glucose levels and diabetes incidence (%) were determined over time. **c** Four GPR40-immune and diabetic RIP-B7.1 mice were injected twice with anti-CD8 antibodies (open circles; *n* = 2) and anti-CD4 antibodies (closed circles; *n* = 2), and blood glucose levels were measured in individual mice for 5 days. **d** Pancreatic sections from representative healthy and diabetic RIP-B7.1 mice were stained for insulin (middle panels) and CD8 T cells (left panels). **e** RIP-B7.1 mice (*n* = 3–4 per group) were injected with pCI/GPR40_1–87,_ pCI/GPR40_74–162_, pCI/GPR40_150–237_ and pCI/GPR40_226–300_ vectors, and diabetes incidence was determined over time. **f** GPR40_187–195_-specific tetramer^+^ CD8 T cells in the pancreata of healthy, pCI-immune (*n* = 3) and pCI/GPR40_150–237_-immune diabetic (*n* = 5) RIP-B7.1 mice were analyzed by FCM. The mean percentage of GPR40_187–195_-specific tetramer^+^ CD8 T cells ± SD is shown. In addition, representative dot blots for each group are shown. **g** PD-L1^−/−^ mice were injected with pCI/GPR40 (*n* = 5; upper panel) or coinjected with pCI/GPR40 and pCI/ppins (*n* = 5; lower panel) and diabetes incidence was determined over time. **h**, **i** PD-L1^−/−^ mice (*n* = 4–5) were injected with pCI (group 1) or pCI/GPR40 (group 2) or coinjected with pCI/GPR40 and pCI/ppins into the left and right tibialis anterior muscle (group 3). **h** Healthy mice were analyzed day 12 post immunization for IFN-γ^+^ GPR40_187–195_-specific CD8 T cells in the spleen and tetramer^+^ CD8 T cells in the pancreas by FCM. **i** At the time of diabetes onset in group 3 (i.e., 4 weeks post immunization), IFN-γ^+^ ppins/(K^b^/A_12–21_)-, and IFN-γ^+^ K^b^/GPR40_187–195_-specific CD8 T cells in the spleen and tetramer^+^ K^b^/GPR40-specific CD8 T cells in the pancreata were determined by FCM. **h**, **i** The mean percentages of IFN-γ^+^ and tetramer^+^ CD8 T cells ± SD are shown. Statistical differences between groups 1 and 2 and between groups 1 and 3 were determined by unpaired Student’s *t* test, and *p* values < 0.05 (*) and < 0.01 (**) were considered significant. ns, not significant

The GPR40 receptor molecule comprises seven transmembrane domains (Fig. 1a). To map MHC I epitopes, we generated four overlapping GPR40 fragment-encoding expression vectors (Fig. 1e). Only pCI/GPR40_150–237_ induced autoimmune diabetes in RIP-B7.1 mice (Fig. 1e). The GPR40_150–237_ fragment contained a GPR_187–195_ sequence in a hydrophobic TMH with two potential K^b^ epitopes, both with anchor residues F at position 5 and L at position 8 or 7/9 [SILL**F**FLP**L** and ILLF**F**LP**L**]. We identified the SILLFFLPL antigenic epitope (Supplementary Fig. S1) and used this peptide to assemble K^b^/GPR_187–195_ tetramers. With this tool, we were able to directly detect K^b^/GPR_187–195_-specific CD8 T cells in the pancreata of pCI/GPR40_150–237_-immune and diabetic RIP-B7.1 mice, but not in the pancreata of pCI-injected healthy control mice (Fig. 1f). Notably, we identified another autoreactive CD8 T-cell response in pCI/GPR40_226–300_-immune and diabetic BALB-RIP-B7.1 mice that was directed against a D^d^/GPR40_236–244_ epitope localized to a different TMH (Fig. 1a; Supplementary Fig. S2).

The injection of pCI/ppins,^6,7^ but not of pCI/GPR40, into PD-L1^−/−^ mice induced autoimmune diabetes (Fig. 1g). We could not detect K^b^/GPR_187–195_-specific IFN-γ^+^ producing effector CD8 T cells in the spleens of healthy mice up to 3 months post immunization (Fig. 1h). However, we detected transient K^b^/GPR_187–195_-specific tetramer^+^ CD8 T-cell populations in the pancreas ~ day 12 postpriming (Fig. 1h). K^b^/GPR_187–195_-specific CD8 T cells were thus primed in PD-L1^−/−^ mice, but they did not acquire a functional IFN-γ^+^ effector phenotype^8^ and were rapidly eliminated in pCI/GPR40-immune PD-L1^−/−^ mice. In contrast, PD-L1^−/−^ mice coinjected with pCI/ppins and pCI/GPR40 vectors developed early and severe autoimmune diabetes that correlated with the presence of circulating IFN-γ^+^ ppins/(K^b^/A_12–21_)-specific CD8 T cells in the spleen (Fig. 1g, i). Most interestingly, we also detected IFN-γ^+^ K^b^/GPR_187–195_-specific effector CD8 T cells in the spleens and tetramer^+^ CD8 T cells in pancreata of these diabetic mice (Fig. 1i). As IFN-γ^+^ K^b^/GPR_187–195_-specific CD8 T cells were detectable in pCI/ppins + pCI/GPR40, mice but not in pCI/GPR40-immune PD-L1^−/−^ mice, their expansion and activation into IFN-γ^+^ effector T cells must be induced by events initiated by ppins/K^b^/A_12–21_-specific CD8 T cells. These findings confirm the crucial role of immunodominant autoreactive CD8 T cells as high-priority targets for novel disease mitigating vaccine strategies.

Our work adds GPR40 to the list of potential autoantigens in immune-mediated T1D. GPR40 is an important component in the fatty acid augmentation of insulin secretion^10^ and is therefore directly linked to the functionality of pancreatic beta cells. As a key sensor of the intraislet milieu, GPR40 may be a novel marker of islet cell autoimmunity and may therefore become a predictive marker for T1D. In particular, the interplay between insulin- and GPR40-directed autoreactivity could also shed more light on the complex events involved in the pathogenesis of immune-mediated diabetes.

## Supplementary information

Supplementary Figures
