# Supplementary material for: A dominant insulin-specific and islet-destructive T-cell response is sufficient to activate CD8 T cells directed against the fatty-acid receptor GPR40
Source: Cell Mol Immunol. 2019 Oct 24;17(6):659–61. doi: 10.1038/s41423-019-0309-y (PMC7264294; doi:10.1038/s41423-019-0309-y)
Supplement: Supplementary file 1 — Supplementary Figures [file 41423_2019_309_MOESM1_ESM.pdf]

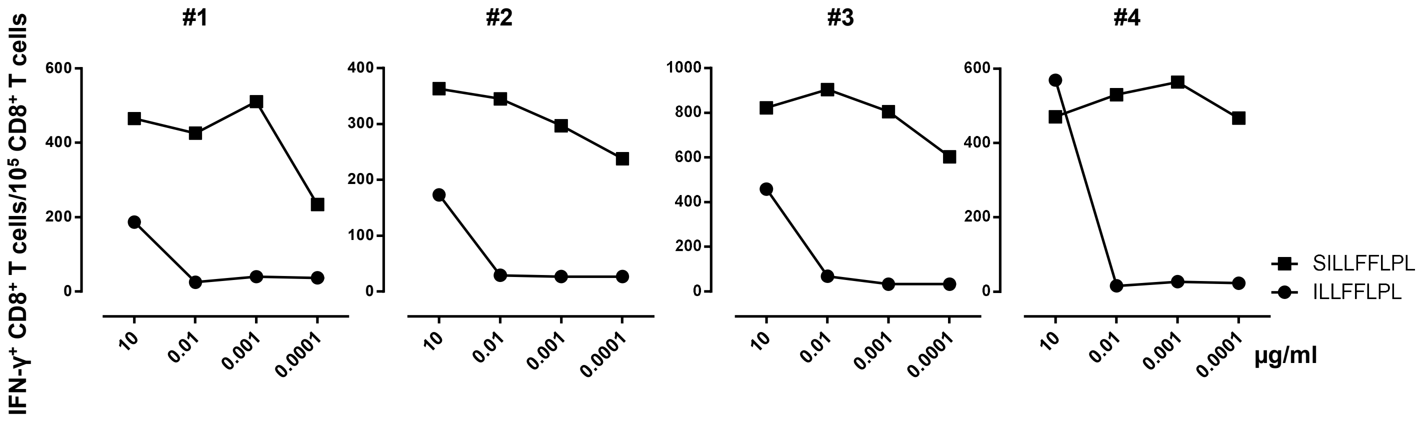

Supplemental Figure S1: Identification of an antigenic GPR40 epitope

Splenic CD8 T cells derived from four individual pCI/GPR40$_{150\text{-}237}$ immune and diabetic RIP-B7.1 tg mice were cultured with titrated doses of SILLFFLPL and ILLFFLPL peptides. Frequencies of IFN-γ^+ CD8 T cells were determined by flow cytometry (FCM) using a BD LSR-II Flow Cytometer. Total numbers of IFN-γ^+ CD8$^+$ T cells per 10^5 CD8$^+$ T cells are shown.

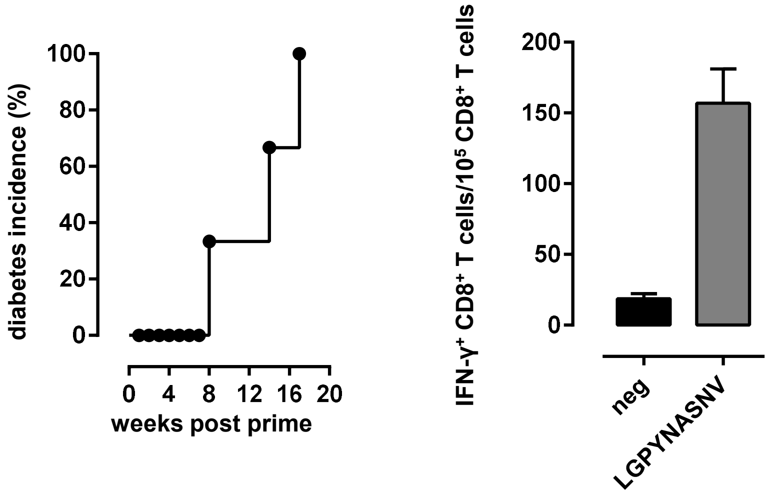

Supplementary Figure S2: Induction of CD8 T cell-mediated autoimmune diabetes in H-2d BALB-RIP-B7.1 tg mice

(a) BALB-RIP-B7.1 tg mice were injected with pCI/GPR40$_{226\text{-}300}$ DNA (n=3) and diabetes incidence was determined over time. **(b)** After diabetes onset, BALB-RIP-B7.1 tg mice were sacrificed and spleen cells were either non-stimulated (neg) or incubated with the predicted D^d/LGPYNASNV (D^d/GPR40$_{236\text{-}244}$) epitope/peptide. Stimulation of IFN-γ production was analyzed by FCM. Means of total numbers of IFN-γ^+ $CD8^+$ T cells per 10^5 $CD8^+$ T cells ±SD are shown.
